# Diagnostic significance of rhythmicity in postural hand tremor

**DOI:** 10.1038/s41598-026-35257-3

**Published:** 2026-01-13

**Authors:** Patricia Weede, Günther Deuschl, Rodger J. Elble, Robin Wolke, Gerhard Schmidt, Gregor Kuhlenbäumer

**Affiliations:** 1 Digital Signal Processing and System Theory, Department of Electrical and Information Engineering, Kiel University, 24143 Kiel, Germany; 2 Department of Neurology, UKSH, Kiel University,Kiel Campus, 24105 Kiel, Germany; 3 Department of Neurology, Southern Illinois University School of Medicine, Springfield, IL 62702 USA

**Keywords:** Tremor, Rhythmicity, Approximate entropy, Essential tremor, Parkinson disease, Physiological tremor, Movement disorders, Neuroscience, Motor control, Central pattern generators

## Abstract

Rhythmicity is an important feature of tremor that is widely viewed as having diagnostic significance. We hypothesized that rhythmicity might be a generic function of tremor severity, reflecting greater oscillatory entrainment of motor pathways. Postural hand tremor and forearm electromyograms were recorded from 49 controls, 78 Parkinson patients, and 133 essential tremor patients. Rhythmicity was quantified in terms of approximate entropy and three measures of cycle-to-cycle frequency variability: tremor stability index, cycle-to-cycle variability, and power spectral bandwidth. Physiological tremor was less rhythmic than Parkinson tremor and essential tremor, but the two pathological tremors did not differ significantly. Hand tremor amplitude and forearm electromyogram-hand tremor coherence were moderate statistical predictors of rhythmicity in both pathological tremors. Adding a 1-kg weight to the hand had little effect on the rhythmicity metrics, except for a moderate reduction in the approximate entropy of physiological tremor. We conclude that these four rhythmicity metrics are not helpful in distinguishing postural tremors in Parkinson disease and essential tremor. The moderate correlations of rhythmicity with tremor amplitude and electromyogram coherence suggest that rhythmicity of pathological tremors is largely a generic reflection of oscillatory neuronal entrainment. Comparisons of tremor rhythmicity in pathological conditions must control for tremor amplitude and electromyogram coherence.

**Supplementary Information:**

The online version contains supplementary material available at 10.1038/s41598-026-35257-3.

## Introduction

Tremor is a common movement disorder and is defined clinically as an involuntary oscillatory movement. Healthy people exhibit physiological tremor (PT) at the threshold of visibility^1^. Pathological tremors are clearly visible and usually symptomatic^2,3^. The International Parkinson and Movement Disorder Society consensus classification of tremor uses two axes of classification: clinical phenomenology (Axis 1) and etiology (Axis 2)^2^. Axis 1 relies primarily on the neurological history and exam, but supportive diagnostic methods using motion transducers, electromyography and additional methods like voice analysis can be included^4^. The most frequent pathological tremors are Parkinson disease tremor (PDT) and essential tremor (ET)^2,3^. Some pathological tremors appear more rhythmic than others, and the degree of rhythmicity is believed to have diagnostic significance.

Rhythmicity refers to cycle-to-cycle variability in frequency, but human perception of rhythmicity could also be influenced by tremor amplitude fluctuations, tremor waveform, and concomitant voluntary and involuntary aperiodic movements^5^. Several quantitative metrics have been used to assess tremor rhythmicity: half-peak power spectral bandwidth (BW)^6^, cycle-to-cycle variability (CTC)^7^, tremor stability index (TSI)^8^, and approximate entropy (ApEn)^9–11^. The diagnostic value of these metrics in tremor classification will depend on the degree to which rhythmicity is determined by differences in underlying pathophysiology. The principal component of physiological tremor is a damped oscillation of an extremity (e.g., hand) in response to random irregularities in muscle contraction and pulsatile cardioballistic forces^12,13^. The frequency of this mechanical-resonant oscillation is a function of limb mass (moment of inertia) and stiffness, and tremor frequency is predictably reduced when a weight is added to the limb. The response of somatosensory receptors to this oscillation is not sufficient to produce a rhythmic modulation of EMG unless stretch-reflex sensitivity is enhanced in some manner, such as by fatigue or the Jendrassik maneuver^14^. By contrast, most pathological tremors, such as PDT and ET, emerge from coherent neuronal oscillation within cerebellothalamocortical motor pathways^12^. The amplitude of PDT and ET is influenced by the frequency–response characteristics of the stretch reflex and joint mechanics, but the frequency of these tremors does not change significantly or predictably with mechanical loading^12^.

Di Biase and co-workers found that the quantification of rhythmicity has diagnostic value in distinguishing PDT and ET^8^. They used accelerometric recordings to compute the interquartile range of cycle-to-cycle change in tremor frequency (TSI) and found that Parkinson rest tremor, re-emergent postural tremor, and non-re-emergent postural tremor were substantially more rhythmic than ET. However, they did not control for tremor amplitude. Pathological tremor amplitude is arguably a reflection of oscillatory entrainment among involved neural networks. Little or no tremor would be expected if participating neurons are oscillating independently of each other. Synchronous entrainment among oscillatory neurons is reflected in the degree of coherence between limb motion and electromyographic (EMG) activity in a participating muscle. Greater entrainment of motoneurons in participating muscles will produce greater coherence (linear correlation squared at a particular frequency) between limb motion and EMG, while muscles contracting independently will have a coherence that is linearly proportional to their fractional participation in limb motion. Extension-flexion of the wrist, for example, is controlled by as many as eight muscles, and coherence will be as low as 1/8 if all muscles are participating equally but independently. EMG with skin electrodes captures the activity of only a small fraction of motor units in a particular muscle, so coherence between EMG and hand motion will approach zero in the absence of widespread motor unit entrainment within and among participating muscles (as in the mechanical-resonant component of physiological tremor).

We hypothesized that tremor rhythmicity is primarily a function of neuronal entrainment among involved neural networks, regardless of diagnosis. In contrast to previous studies, we used a comprehensive set of rhythmicity metrics to assess commonalities and differences in large cohorts. We used tremor amplitude and its coherence with EMG as surrogate measures of tremorogenic neuronal entrainment. Specifically, we performed accelerometric hand tremor (HT) and electromyographic muscle tremor (MT) recordings to determine the extent to which the rhythmicity of PT, PDT and ET is determined by HT amplitude and MT-HT coherence versus diagnosis. We added weight to the hand to examine the effect of increased muscle contraction and lowered mechanical resonant frequency in all three subject groups.

## Methods

### Study population

Consecutive patients underwent a standardized tremor analysis at the Department of Neurology, Kiel University, and healthy controls were recruited from hospital personnel, students, and their relatives. Based on published diagnostic clinical criteria^3^, the dataset (*n* = 395) consisted of 52 healthy adults, 210 patients with ET, and 131 patients with PD and postural tremor, after excluding 12 individuals with poor quality recordings. To clearly separate physiological and pathological postural tremors, we removed all patients with a diagnosis of ET (*n* = 77) or Parkinson disease (*n* = 53) lacking a detectable MT in either the unloaded or loaded condition. We therefore excluded mildly abnormal tremors with no MT in the muscle studied (extensor carpi radialis brevis). We also excluded three controls who had a detectable MT in the unloaded or loaded condition to exclude enhanced physiological tremor or undiagnosed pathological tremor.

Informed consent was obtained from all participants. Individuals with legal guardians or underage individuals were not included in the study. This human research complied with all relevant national regulations and institutional policies in accordance with the tenets of the Helsinki Declaration and was approved by the authors’ Institutional Review Board (Ethics Committee of the Kiel Medical Faculty).

### Study protocol

Postural HT was recorded from each upper limb for 30 s while subjects sat in a chair with forearm supported by an armrest and the hand extended horizontally. The hand and wrist protruded beyond the armrest with the palm facing downward. The limb with greatest tremor was selected for analysis.

Commercially available equipment was used (Nicolet EDX EMG System, Natus Medical Incorporated). Uniaxial accelerometers with a sensitivity of 1 milligravity and range up to 20 gravities were attached to the dorsal surface of both hands, near the distal third metacarpal. MT was recorded using surface EMG with commercial Ag/AgCl electrodes over the extensor carpi radialis brevis. The EMG sensitivity was 10 nV, with a range of 50 mV. The EMG and accelerometer signals were digitized synchronously at 800 Hz. Hand tremor was recorded with and without a 1-kg weight on the distal metacarpus^12^. Tremor measurements were conducted by our trained technicians during the morning, and caffeine intake and sleep hygiene were not controlled. Data were completely anonymized, and treatment data were not available.

### Tremor analysis

We used MATLAB (https://de.mathworks.com/products/matlab.html) to analyse accelerometer and EMG recordings. Details are described in the following.

#### Tremor frequency

Power spectral density was computed using the Welch method in MATLAB. With a sampling frequency of 800 Hz and a signal duration of 30 s, the total length of each signal was 24,000 samples. The signal was divided into segments with a length of 2048. With an overlap of 50%, approximately 23 segments were considered in the calculation of power spectral density. Each segment was Hann windowed. Zero padding was used to achieve estimates of spectral peak frequency and half-peak power frequency with a resolution of 0.024 Hz. A modified harmonic product spectrum algorithm was used to compute the fundamental tremor frequency in the power spectrum^6^. This tremor frequency was defined as significant if the corresponding spectral peak was larger than the adjacent minima plus two standard deviations^6,12,15^.

#### Hand tremor (HT)

Spectral analysis of acceleration recordings (time series) was performed without additional preprocessing. We defined HT as present when an accelerometric spectral peak was statistically significant^6,12,15^. HT was present in all participants, and the acceleration time series from controls and patients with PD and ET consist almost entirely of tremor^12^. Therefore, tremor amplitude was calculated as the root mean square of the acceleration recording. Hand tremor amplitude is expressed in units of meters per second squared (m/s^2^).

#### Muscle tremor (MT)

MT describes the rhythmic modulation of EMG amplitude. The signal from electrodes placed over the extensor carpi radialis brevis muscle was used in the analysis. We pre-processed the EMG signal by performing amplitude demodulation^6,15,16^. First, we applied a high-pass filter with a cut-off frequency of 50 Hz to remove low-frequency artefacts. The resulting signal was full-wave rectified and low-pass filtered with a cut-off frequency of 30 Hz. This cut-off frequency captures all significant activity in PT, PDT and ET. We defined MT as present when the EMG power spectral peak was statistically significant and within ± 1 Hz of the hand tremor frequency^6,12,15^. Muscle tremor amplitude is expressed in units of microvolts (µV) and was calculated as the root mean square of the amplitude-demodulated EMG.

#### Coherence

Magnitude-squared coherence between amplitude-demodulated EMG and accelerometry was computed with MATLAB function mscohere(). The values of coherence range from zero to one. High coherence at a particular frequency occurs when amplitude-demodulated EMG and hand acceleration rise and fall together at a particular frequency. In this study, we report MT-HT coherence at the tremor frequency.

The 99% confidence level for coherence exceeding 0 is $\text{1 - }{(1 - \alpha )}^{\frac{1}{L-1}}$, where $\alpha =0.99$ and *L* as the number of overlapping segments used for spectral averaging^17,18^. With a signal length of 30 s $\bullet$ 800 samples/s = 24,000 samples, a segment size of 2048 without zero padding, and an overlap of 50%, the rounded *L* is 24,000/2048/0.5 = 23 segments. With these parameters, the 99% confidence interval is 0.188.

Noise estimation was used to enhance our ability to compute coherence between the signals independent of noise. This estimation is based on a multiplicative time-constant approach in which the spectrum is multiplied by a factor slightly larger or slightly smaller than one depending on the spectrum itself^19^. With this approach it is possible to estimate a lower limit of the spectrum as noise. A Wiener filter is used to reduce the influence of noise on the signals before coherence analysis. This filtering method minimizes the mean squared error between the original signal and the filtered version and is important to correct the noise-induced attenuation of the signal amplitude^20^.

#### Rhythmicity metrics

**Fig. 1:**
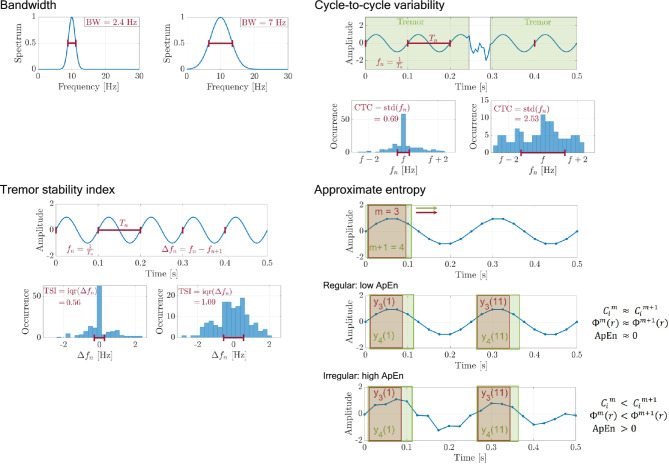
Principles of computing the four rhythmicity metrics. Bandwidth (BW) is the width of the tremor power spectral peak at half peak power. Cycle-to-cycle variability (CTC) is the standard deviation of sequential instantaneous frequency, and the tremor stability index (TSI) is the interquartile range of sequential frequency differences. The approximate entropy algorithm uses short sliding windows to identify local patterns of length *m*, and the fraction of similar patterns is averaged across the signal to quantify the overall predictability (*Φ*). Lower *Φ* indicates greater irregularity, producing higher ApEn values. Results are shown for hypothetical time series with low and high rhythmicity/predictability.

HT rhythmicity was quantified with established metrics in the time (cycle-to-cycle variability, tremor stability index, and approximate entropy) and frequency (bandwidth) domains. These metrics are illustrated in Fig. 1.

##### Bandwidth (BW, Fig. 1)

BW is defined as the width of a tremor spectral peak at half-peak spectral power, and this was calculated only for statistically significant peaks in HT and MT ^6,15^. BW is inversely related to rhythmicity and becomes increasingly narrow with decreasing frequency variability. An oscillation with no frequency variability produces an infinitely narrow spectral peak or spike at the fundamental frequency of oscillation.

##### Cycle-to-cycle variability (CTC, Fig. 1)

The signals were filtered digitally for this measurement using a 10^th^-order zero-phase Butterworth low-pass filter (i.e., filtering with a 5^th^-order Butterworth filter in both temporal directions). A cut-off frequency of 15 Hz was selected to filter high-frequency activity above the range of tremor^7^. After normalization with the mean amplitude, cycle-to-cycle frequency values were calculated as the inverse of consecutive time differences between zero crossings of the signal with a positive slope. Interruptions in tremor were discarded by removing sections in which the current cycle frequency differed more than 2 Hz from the previous and subsequent frequencies^7^. The remaining sections are defined as containing continuous tremor. CTC is defined as the standard deviation of the serial cycle-to-cycle tremor frequencies.

##### Tremor stability index (TSI, Fig. 1)

TSI is another measure of the variability in cycle-to-cycle tremor frequency^8^. The time signals are first detrended and digitally filtered using a 10^th^-order zero-phase Butterworth bandpass filter. The cut-off frequencies are defined in terms of the spectral peak frequency: ${f}_{cutoff}={f}_{peak}\pm 2 {\mathrm{Hz}}$. TSI is the interquartile range of the sequential differences in cycle-to-cycle frequencies. The lowest possible value of TSI is zero for an oscillation with no frequency variability, but TSI is always greater than zero (typically ≥ 0.1) because no tremor is perfectly rhythmic. The highest value of TSI is limited by the fact that increasing frequency variability will broaden the spectral peak BW to an extent that peak amplitude is no longer statistically significant. Practically speaking, we observed maximum TSI values of approximately 2 Hz for accelerometry.

##### Approximate entropy (ApEn, Fig. 1)

ApEn is a measure of predictability in a time series and has been used as a rhythmicity metric for tremor^9–11^. ApEn is influenced by cycle-to-cycle variability in frequency and waveform. A value of zero denotes perfect predictability, as for a sine wave with no noise, while higher values indicate a more random time series that is less predictable.

MATLAB function approximateEntropy() was used to compute ApEn of accelerometer recordings that were low-pass filtered at 30 Hz (10th-order Butterworth). Parameters were *m* = 5 and *r* = 0.125 · SD. Here, *m* specifies the length of a sliding pattern window, *r* sets the similarity tolerance, *C* is the fraction of matching patterns, and *Φ* is the average predictability across patterns (Fig. 1).

Published studies of biological signals including tremor recommended a window size *m* = 3 and a tolerance *r* = 0.2 · SD^9,10,21^. However, simulations with perfectly rhythmic sinusoidal signals of the same duration (30 s), sampling frequency (800 Hz), and frequency range (3–10 Hz) revealed a strong dependence of ApEn on oscillation frequency using the published recommended values of *m* and *r*. Therefore, we performed a systematic grid search of 1 ≤ *m* ≤ 10 and 0.1 · SD ≤ *r* ≤ 0.25 · SD using both noisy and ideal, perfectly rhythmic sinusoidal time series. This process was aimed at identifying the optimal values that met two key criteria: the chosen parameters should minimize the dependence of ApEn on the signal fundamental frequency, and the parameters must still be sensitive enough to detect variability in the signal waveform and frequency. Based on these criteria, we found that a window size of *m* = 5 and tolerance of *r* = 0.125 · SD were optimal. These specific values effectively eliminated the dependence of ApEn on the frequency of ideal sinusoidal signals but still detected variation in frequency and waveform.

### Statistical analysis

We used R version 4.3.0 for all statistical calculations and the package ggplot2 (https://ggplot2.tidyverse.org/) for all figures except Fig. 1^22^. The distributions of variables were assessed with visual histogram analysis. HT and MT amplitudes (Amp_HT and Amp_MT), TSI, CTC and BW were log_2_-transformed to achieve approximate normality because regression analyses are improved by using normally distributed variables and biological signals often exhibit log-linear or log–log relationships. The distribution of BW was extremely right skewed and was not approximately normal even after log_2_-transformation. The Wilcoxon test (WT) was used for most paired-test comparisons, but we always performed t-tests in addition to WT. The t-test results are not reported but confirmed the results of WT in all instances. We used linear models with type I sums of squares (R default) and Pearson correlations for all calculations involving linear models and noted when linear models were not adequate. R-squared (R^2^) values for the linear models are adjusted R^2^ values. The Lindeman-Merenda-Gold (LMG) method was implemented in “relaimpo” for predictor importance analysis in R^23,24^. The effects of weight loading were quantified using Cohen’s d with correction for paired samples (effect size: cohens_d (paired = T))^25^ because in contrast to simple fold-difference, it takes variability into account. We used a significance threshold of *p* < 0.01 to reduce type II errors and diminish the detection of very weak associations in this comparatively large sample. We used the variance inflation factor (VIF) as an indicator of collinearity between independent variables in multiple linear regressions. The VIF’s of all variables in the multiple linear regression in this and all subsequent sections were below three and, in most cases, between one and two, indicating the lack of excess collinearity.

Correlations are interpreted as r = 1 perfect, 0.7 ≤ r < 1 strong, 0.3 ≤ r < 0.7 moderate, 0 < r < 0.3 weak and r = 0 no linear relationship. Negative values are interpreted similarly but in the inverse direction. R^2^ is interpreted using the same scale. Similarly, the thresholds for Cohen’s d interpretations are d = 0 no effect, d = 0.2 small effect, d = 0.5 medium effect, d = 0.8 large effect, and d ≥ 1.2 very large effect.

## Results

### Demographics

Demographic characteristics of the control and patient cohorts are summarized in Table 1. ET and Parkinson patients were significantly older than controls, but age did not differ between ET and Parkinson patients. The control group contained significantly more females than the pathological tremor groups, and the disease duration was significantly longer in ET compared to PDT.

**Table 1:** Demographic characteristics of the control and patient cohorts. NA: not applicable.

	Physiological tremor (PT) (n = 49)	Parkinson disease tremor (PDT) (n = 78)	Essential tremor (ET) (n = 133)	p-value PT vs. PDT	p-value PT vs. ET	p-value PDT vs. ET	Test
Age (years, mean ± SD)	43 ± 21	56 ± 9	56 ± 12	0.000	0.000	0.964	t test
Female n (%)	29 (59%)	31 (40%)	37 (28%)	0.044	0.000	0.093	Fisher test
Disease duration (years, mean ± SD)	NA	11 ± 7	22 ± 14	NA	NA	0.000	t test

### Tremor metrics

**Fig. 2:**
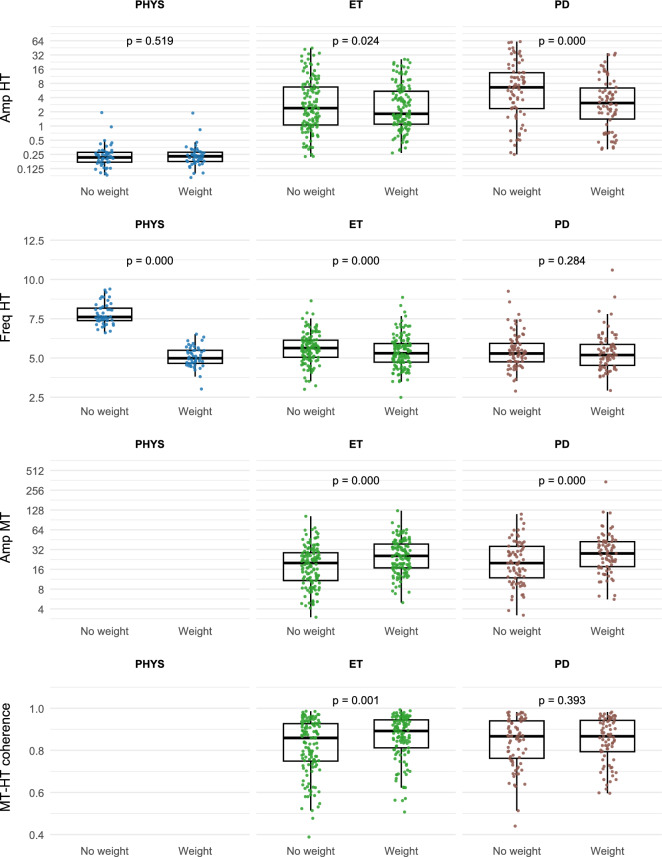
Tremor metric distributions with and without weight loading. Hand tremor (HT) and muscle tremor (MT) amplitudes (Amp), hand tremor frequency (Freq: Hz), and MT-HT coherence are shown for the three diagnostic groups: physiological tremor (PHYS), essential tremor (ET) and Parkinson disease (PD). Wilcoxon test p values are shown for comparisons of data with and without weight loading. Hand tremor in meters per second squared (m/s^2^) and muscle tremor in microvolts (µV) are plotted on a log_2_ scale.

All participants had a significant HT frequency peak. Physiological tremor, by design, had no MT or MT-HT coherence. The distributions of tremor metrics with and without weight loading are shown in Fig. 2. HT and MT amplitudes (Amp_HT and Amp_MT) were approximately normal after log_2_-transformation. MT-HT coherence was highly skewed toward values of 1, and this did not improve with log transformation.

One-way ANOVAs were performed looking for group (diagnosis) differences in log_2_Amp_HT, log_2_Amp_MT, Freq_HT, and MT-HT coherence. PT had a much lower mean log_2_Amp_HT and higher Freq_HT than the two pathological tremors. Mean log_2_Amp_HT was greater for PDT than ET, but these two tremors did not differ in frequency, log_2_Amp_MT or MT-HT coherence. These results are summarized in Table 2.

**Table 2:** One-way ANOVA of tremor metrics versus diagnosis. ET: essential tremor, PDT: Parkinson disease tremor, PT: physiological tremor. HT: hand tremor (meters per second squared: m/s^2^). MT: muscle tremor (microvolts: µV).

Metric	ANOVA			Mean ± SD			Post hoc comparison p values	
	F	dof	p	ET	PDT	PT	PDT–ET	PT–(ET,PD)
log_2_Amp_HT (m/s^2^)	108.5	2, 257	< 0.001	1.45 ± 1.84	2.43 ± 2.01	− 2.12 ± 0.78	< 0.001	< 0.001
Freq_HT (Hz)	112.3	2, 257	< 0.001	5.61 ± 0.94	5.43 ± 1.11	7.80 ± 0.69	0.181	< 0.001
log_2_Amp_MT (µV)	1.43	1, 209	0.234	4.16 ± 1.01	4.33 ± 1.08	—	—	—
MT-HT coherence	1.26	1, 209	0.264	0.82 ± 0.13	0.84 ± 0.12	—	—	—

### Rhythmicity metrics

**Fig. 3:**
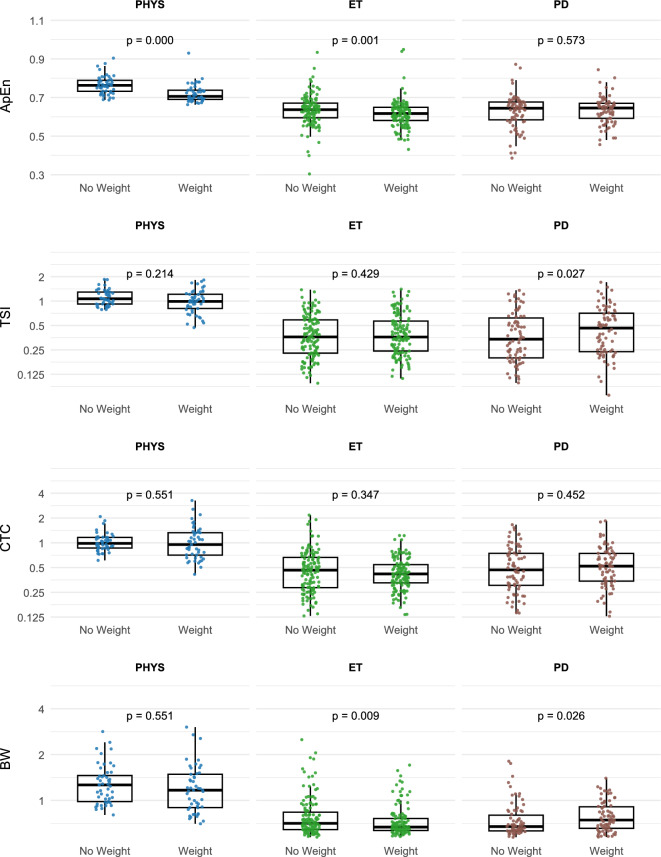
Rhythmicity metric distributions with and without weight loading. Distributions of approximate entropy (ApEn), tremor frequency stability index (TSI), cycle-to-cycle frequency variability (CTC) and tremor bandwidth (BW) are shown for hand tremor (HT) in the three diagnostic groups: physiological tremor (PHYS), essential tremor (ET) and Parkinson disease (PD). Wilcoxon test p values are shown for comparisons of data with and without weight loading. TSI, CTC and BW are plotted on a log_2_ scale and have units of Hz. ApEn is dimensionless.

Distributions of the rhythmicity metrics with and without weight loading are shown in Fig. 3. ApEn was approximately normally distributed in all diagnostic groups, but TSI, CTC and BW were very skewed and required log_2_-transformation. BW was extremely right skewed and was not sufficiently normalized even after log_2_ transformation.

One-way ANOVAs were performed to determine if diagnostic group differences existed for the four rhythmicity metrics: ApEn, log_2_ TSI, log_2_ CTC and log_2_ BW. All four metrics were significantly greater in the controls (p < 0.001) but were statistically equal in ET versus PD. These results were confirmed with Kruskal–Wallis and Dunn post hoc testing. ANOVA statistics are provided in Table 3.

**Table 3:** One-way ANOVA of rhythmicity metrics versus diagnosis. Approximate entropy (ApEn), tremor frequency stability index (TSI), cycle-to-cycle frequency variability (CTC) and tremor bandwidth (BW) of hand tremor (HT) are shown for the three diagnostic groups: physiological tremor (PT), essential tremor (ET) and Parkinson disease tremor (PDT). TSI, CTC and BW are in Hz. ApEn is dimensionless.

Metric	ANOVA		Mean ± SD			Post hoc comparison p values		
	F(2,257)	p (ANOVA)	ET	PDT	PT	PDT—ET		PT—(ET,PDT)
ApEn_HT	60.9	< 0.001	0.63 ± 0.08	0.63 ± 0.09	0.77 ± 0.05	0.551		< 0.001
log_2_TSI_HT	73.4	< 0.001	− 1.45 ± 0.86	− 1.49 ± 1.01	0.15 ± 0.31	0.781		< 0.001
log_2_CTC_HT	43.4	< 0.001	− 1.17 ± 0.82	− 1.08 ± 0.90	0.01 ± 0.35	0.427		< 0.001
log_2_BW_HT	68.9	< 0.001	− 0.37 ± 0.41	− 0.44 ± 0.36	0.35 ± 0.42	0.203		< 0.001

We also examined the effect of dividing TSI, CTC and BW by tremor frequency to produce normalized values of rhythmicity. Normalizing TSI, CTC and BW did not improve their ability to discriminate ET, PDT, and PT.

### Univariate correlations between tremor metrics with rhythmicity

The rhythmicity metrics and tremor metrics were examined in a Pearson correlation matrix for each diagnostic group and for PD and ET combined (supplementary Tables S1-4). For physiological tremor, there was no significant correlation between log_2_Amp_HT and Freq_HT, and these tremor metrics did not correlate with any of the four rhythmicity metrics. However, the rhythmicity metrics were moderately correlated with each other except for a strong correlation between log_2_TSI_HT and log_2_CTC_HT and no significant correlation between ApEn_HT and log_2_BW_HT.

For ET and PDT, log_2_Amp_HT was negatively correlated with Freq_HT as described previously for ET^26^. The rhythmicity metrics were weakly to moderately correlated with the four tremor metrics, except for a lack of significant correlation between log_2_BW_HT and log_2_Amp_MT and between log_2_CTC_HT and Freq_HT. The tremor metrics were weakly to moderately correlated with each other except for a lack of significant correlation between Freq_HT and MT-HT coherence. The rhythmicity metrics were moderately to strongly correlated with each other.

The linear univariate relationships between the four rhythmicity metrics and four tremor metrics were computed for ET and PDT and are summarized in Table 4. All tremor metrics modelled poorly with log_2_BW_HT. The ET-PDT slope comparisons had *p* values < 0.05 in only three of 16 instances, and in no instance was p less than the Bonferroni *p* value of 0.003. For all rhythmicity metrics, R^2^ was greatest when the tremor metric predictors were log_2_Amp_HT and MT-HT coherence, even though coherence was range-restricted to values near one. The linear model was not improved by coherence transformations and polynomial terms.

**Table 4:** Univariate linear regressions of rhythmicity metrics vs tremor metrics for postural hand tremor (HT). The slopes for Parkinson disease tremor (PDT) and essential tremor (ET) are compared statistically. The rhythmicity metrics are approximate entropy (ApEn_HT), log2 tremor stability index (log_2_TSI_HT), log_2_ cycle-to-cycle variability (log_2_CTC_HT), and log_2_ bandwidth (log_2_BW_HT). Tremor metrics are log_2_ amplitude of HT (log_2_Amp_HT: meters per second squared), log_2_ amplitude of muscle tremor (log_2_Amp_MT: microvolts), HT frequency (Freq_HT: Hz), and MT-HT coherence. TSI, CTC and BW are in Hz. ApEn is dimensionless.

Rhythmicity metric	Tremor metric	Slope (± SE)		R^2^	p values
		ET	PDT	(ET, PDT)	ET—PDT slope
ApEn_HT	log₂Amp_HT	−0.023 ± 0.003	−0.024 ± 0.004	0.29, 0.31	0.781
ApEn_HT	log₂Amp_MT	−0.015 ± 0.007	−0.032 ± 0.008	0.04, 0.16	0.105
ApEn_HT	Freq_HT	0.021 ± 0.007	0.018 ± 0.009	0.06, 0.05	0.798
ApEn_HT	MT-HT coherence	−0.257 ± 0.047	−0.329 ± 0.077	0.19, 0.19	0.414
log₂TSI_HT	log₂Amp_HT	−0.228 ± 0.036	−0.328 ± 0.043	0.24, 0.43	0.076
log₂TSI_HT	log₂Amp_MT	−0.129 ± 0.074	−0.467 ± 0.092	0.02, 0.25	**0.005**
log₂TSI_HT	Freq_HT	0.074 ± 0.080	0.209 ± 0.101	0.01, 0.05	0.282
log₂TSI_HT	MT-HT coherence	−2.338 ± 0.538	−5.028 ± 0.806	0.13, 0.34	**0.006**
log₂CTC_HT	log₂Amp_HT	−0.200 ± 0.035	−0.263 ± 0.041	0.20, 0.35	0.245
log₂CTC_HT	log₂Amp_MT	−0.096 ± 0.070	−0.403 ± 0.083	0.01, 0.24	**0.006**
log₂CTC_HT	Freq_HT	0.012 ± 0.076	0.146 ± 0.091	0.00, 0.03	0.252
log₂CTC_HT	MT-HT coherence	−2.283 ± 0.509	−3.131 ± 0.805	0.13, 0.17	0.362
log₂BW_HT	log₂Amp_HT	−0.078 ± 0.018	−0.044 ± 0.020	0.12, 0.06	0.228
log₂BW_HT	log₂Amp_MT	−0.045 ± 0.036	−0.037 ± 0.038	0.01, 0.01	0.884
log₂BW_HT	Freq_HT	0.086 ± 0.038	0.065 ± 0.036	0.04, 0.04	0.701
log₂BW_HT	MT-HT coherence	−0.742 ± 0.268	−1.150 ± 0.325	0.06, 0.14	0.362

### Determinants of hand tremor rhythmicity in pathological tremors

**Fig. 4:**
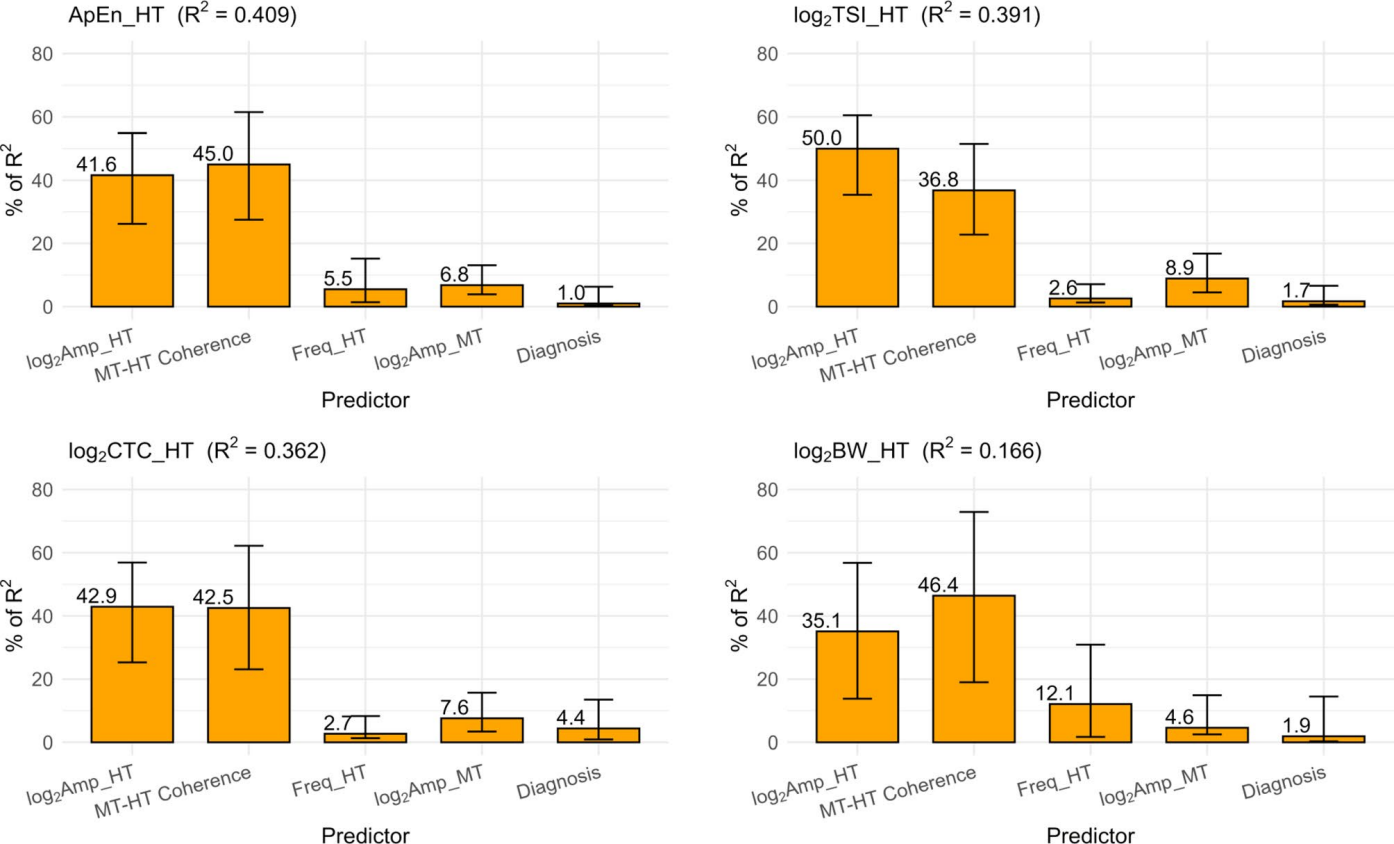
Lindeman-Merenda-Gold analysis of four tremor metrics and diagnosis as predictors of rhythmicity. Rhythmicity metrics are approximate entropy (ApEn_HT), tremor stability index (TSI_HT), cycle-to-cycle variability (CTC_HT), and bandwidth (BW_HT) of hand tremor (HT). Tremor metrics predictors are hand tremor amplitude (Amp_HT), muscle tremor amplitude (Amp_MT), hand tremor frequency (Freq_HT), and MT-HT coherence. Diagnosis factors are Parkinson disease and essential tremor. The 95% confidence limits were computed with the bootstrap method.

We examined the multivariate linear relationship of each rhythmicity metric to the four tremor metrics, with diagnosis as a factor. To assess predictor importance and overcome the problem of shared variance, we computed relative predictor importance using the LMG method, which averages the sequential sums of squares over all orderings of the predictors and thereby partitions the unique and shared variance^24^. The results are summarized in Fig. 4 where LMG importance is expressed as the percentage of R^2^ explained by a tremor metric predictor. Consistent with the preceding univariate analyses, log_2_Amp_HT and MT-HT coherence were the main predictors, and diagnosis (ET vs PD) made no significant contribution.

### Effect of weight loading on hand tremor rhythmicity metrics

Weight loading is known to reduce the frequency of physiological tremor in proportion to the size of the weight, while loading has little or no effect on the frequency of ET and PDT^12^. This was confirmed in the present study (Fig. 2). Freq_HT decreased strongly (mean ± SD = −2.73 ± 0.66 Hz) with weight loading in PT (d = −4.26, *p* ≤ 0.0001, WT paired), decreased by a small but statistically significant amount in ET (−0.22 ± 0.55 Hz; d = −0.40, *p* ≤ 0.0001, WT paired), but did not change significantly in PDT (d = −0.08, *p* = 0.284, WT paired). Amplitude of HT decreased statistically in PDT (d = −0.76, *p* ≤ 0.0001, WT paired) while the amplitude of MT increased in PDT (d = 0.67, *p* ≤ 0.0001, WT paired) (Fig. 2).

ApEn decreased significantly with weight loading in PT (−0.04 ± 0.06 d = −0.71, p ≤ 0.0001, WT paired), and this was associated with a −2.73 Hz decrease in frequency. We considered the possibility that the change in ApEn was due to residual algorithmic dependence of ApEn on frequency. However, there was no significant correlation between ApEn_HT and Freq_HT without (r = 0.074) or with (r = 0.107) loading, nor was there a correlation between the change in ApEn_HT and change in Freq_HT with loading (r = 0.120) (*p* > 0.4 for all correlations).

All other changes in TSI, CTC and BW were small or insignificant. ApEn decreased with weight loading in ET (d = −0.20, *p* = 0.0001, WT paired) but not in PDT (d = 0.07, *p* = 0.585, WT paired). Small but statistically significant changes were observed for TSI in PDT (d = 0.31, *p* = 0.009, WT paired) and for BW in ET (d = −0.25, *p* = 0.009, WT paired).

## Discussion

We systematically analysed the statistical association of four measures of rhythmicity (ApEn, TSI, CTC, and BW) with four hand tremor metrics (Amp_HT, MT-HT coherence, Freq_HT, and Amp_MT) in normal controls (N = 49) and in patients with PD (N = 78) or ET (N = 133). We specifically examined whether ET and PDT differ in rhythmicity and if rhythmicity is a function of tremor metrics. We found that PT is less rhythmic than ET and PDT, but these two pathological tremors did not differ. Tremor amplitude (Amp_HT) and EMG-tremor coherence (MT-HT coherence) were moderate predictors of rhythmicity in the two pathological tremors but not in physiological tremor. We did not include controls with enhanced physiological tremor, which we hypothesize would exhibit rhythmicity that is significantly correlated with Amp_HT and MT-HT coherence. This hypothesis should be examined in future studies.

The rhythmicity metrics in this study were moderately to strongly correlated with each other. However, computational differences in these metrics are noteworthy. BW is defined as the width of a tremor spectral peak at half-peak spectral power. Frequencies outside this frequency band do not contribute to this measure of rhythmicity^6,15^. TSI is computed after the tremor time series is bandpass filtered at the peak frequency ± 2 Hz, and CTC is computed after low-pass filtering at 15 Hz^7,8^. Accelerometry was lowpass filtered at 30 Hz, for ApEn analysis. Thus, the frequency content of the filtered time series is not the same for TSI, CTC, and ApEn, and differences in filtering will influence estimates of variation in frequency and waveform. ApEn is a measure of regularity (predictability) in frequency and waveform, while BW, TSI and CTC are relatively pure measures of variation in frequency^21^.

Other methodological considerations should be considered when choosing among the four rhythmicity metrics in this study. ApEn exhibits a known methodological bias such that ApEn decreases in proportion to oscillation frequency^27,28^. This bias is a function of the algorithm parameters (m, r, τ) and the range of frequencies in the sampled data. We optimized these parameters for our data using a computer simulation with artificial perfectly-rhythmic sinusoids. We were able to reduce the correlation between frequency and ApEn from 0.732 to 0.246 over the frequency range (3–9 Hz) of our physiological and pathological tremors. Investigators should be aware of this bias, which also exists for sample entropy^27,28^.

Dividing TSI, CTC and BW by tremor frequency produces normalized values of rhythmicity. Normalizing TSI, CTC and BW did not improve their ability to discriminate ET from PDT. However, this approach might have greater impact when comparing tremor disorders with widely disparate frequencies, such as myorhythmia and primary orthostatic tremor^18^.

The rhythmicity of tremor is frequently assessed by neurological examination and is often discussed in terms of “jerkiness”^5^. Waveform and frequency variability, waveform shape (e.g., sinusoidal vs triangular, sawtooth or square wave), intermittency in oscillation, and coexistence of involuntary aperiodic movements (e.g., myoclonus and dystonia) could individually or collectively produce the impression of irregularity or jerkiness in tremor^5^. It is unclear which properties of tremor contribute most to the visual impression of jerkiness or arrhythmicity^18^. It seems likely that cycle-to-cycle variability in tremor frequency (a.k.a., jitter) is imperceptible unless it is quite large, but to our knowledge, this has not been formally studied. Therefore, the relevance of our results to the clinical perception of jerkiness is unclear. Intermittent fluctuations in jitter, interruptions in tremor, and coexistent aperiodic movements are probably more perceptible as jerkiness than the relatively constant levels of frequency variability quantified in our study. More research is needed to identify the kinematic features of tremor that influence the human perception of rhythmicity.

We analysed EMG from the extensor carpi radialis brevis only. MT-HT coherence is squared linear correlation between MT and HT at the tremor frequency. If coherence between one muscle and hand tremor is almost 1, this muscle is either the only muscle involved in the tremor (i.e., the only muscle contributing to tremor variance), or this muscle is strongly coherent with all other involved muscles, as demonstrated previously^29^. High coherence among muscles controlling the wrist can only occur if there is high coherence (oscillatory entrainment) of the involved motor pathways, which is known to occur in PDT and ET^12^.

LMG analysis demonstrated that the tremor amplitude and EMG-hand tremor coherence were comparable predictors of ApEn, TSI, CTC, and BW, although BW did not model well using linear regression. Significant correlations between tremor amplitude and the four rhythmicity metrics were not found in PT. This is expected because unenhanced PT is a stochastic process in which rhythmicity is determined solely by biomechanical properties of the wrist/hand^30–33^. The relatively minor contribution of muscle tremor amplitude in PD and ET may be explained by the factors that limit EMG amplitude and its relation to muscle force, including muscle location, muscle size, the impedance of surrounding skin and subcutaneous fatty tissue, the impedance of EMG electrodes, and the nonlinear relationship between EMG and muscle force^34^.

Our results suggest that large-amplitude, highly coherent tremors tend to be highly rhythmic, regardless of diagnosis. Most pathological tremors are driven by rhythmic coherent muscle contraction produced by coherent oscillation in cerebellothalamocortical motor pathways, although resonance with the segmental mechanical-reflex system may contribute^12,33^. Other studies found differences in rhythmicity metrics (particularly TSI and CTC) between patients and controls^9,35,36^, between dystonic tremor and ET^7,37^, and between tremors in chronic inflammatory demyelinating neuropathy (CIDP), dystonia and ET^38^. One large study found that TSI of HT but not MT separated PDT and ET with high confidence^8^. This report conflicts with our finding that the rhythmicity metrics TSI, ApEn, CTC, and BW do not differ significantly between PDT and ET. We have shown that HT amplitude and MT-HT coherence are critical predictors of rhythmicity and must be considered in the interpretation of rhythmicity differences among diagnostic groups. HT amplitude and MT-HT coherence were not adequately considered in prior diagnostic studies.

Weight loading is often used for diagnostic purposes because it strongly and predictably reduces the frequency of PT but does not consistently alter the frequency PDT and ET^6,12^. This was confirmed in our study. Weight loading will exert a low-pass filtering effect on any tremor by lowering the natural frequency (Eigenfrequency) of the wrist, thereby attenuating higher-frequency noise and harmonics. This intervention substantially reduced ApEn in patients with PT, modestly in ET, and not at all in PDT. In contrast, weight loading had no significant effect on the pure rhythmicity metrics (TSI, CTC, and BW) across PT, ET, and PDT. These findings suggest that the rhythmicity and waveform structure of ET and PDT are relatively insensitive to the mechanical filtering properties of the wrist.

The most important strength of our study is the large dataset of patients and controls, which surpasses that of any previous study^35,36^. Our patients were evaluated in a tertiary referral centre with special emphasis on movement disorders, and tremor was assessed with a standardized protocol. A limitation of our study is the anonymity of the data, which precludes us from returning to the patients’ records in search of additional data, such as medications, that might help with data interpretation. Medications reduce rhythmicity of PD in proportion to the reduction in tremor amplitude^39^. We hypothesize that a similar relationship exists for ET, but the medications for ET are often ineffective, making this hypothesis difficult to test. Another limitation is that recruitment from a tertiary referral centre will always be biased toward more severe phenotypes. The exclusion of ET/PDT with no detectable MT might have introduced a bias towards more severe pathological tremors or towards tremors involving the extensor carpi radialis. Recordings from multiple wrist extensor and flexor muscles would allow a more comprehensive assessment of MT-HT coherence and its relationship to rhythmicity and would aid in identifying enhanced PT (i.e., PT with MT). We excluded controls with significant MT-HT coherence in order to exclude enhanced physiological tremor, but it is possible that some of our controls had tremor-related EMG activity in muscles not recorded. Controls with coherent MT have tremor amplitudes that are more than 2 × control tremor without coherent MT^40^. The inclusion of controls with coherent EMG activity (i.e., enhanced physiological tremor) would likely increase the correlation between tremor amplitude and rhythmicity, as previously found by Sturman and coworkers^41^. Therefore, it seems likely that the limitations in our study had no significant impact on the principal results, but the exact values of the metrics are likely to be different in other samples.

## Conclusions

1. Unenhanced physiological postural hand tremor is less rhythmic than postural tremor in Parkinson disease and essential tremor.
2. The rhythmicity of Parkinson and essential postural tremors is a moderate correlate of tremor amplitude and muscle-hand tremor coherence, which arguably reflect the strength of central oscillatory neuronal entrainment. Rhythmicity does not distinguish Parkinson disease tremor from essential tremor when one controls for differences in tremor amplitude.
3. Weight loading the hand reduces ApEn of physiological tremor, probably through a low-pass mechanical filtering effect, but it has no significant effect on the other rhythmicity metrics. Weight loading has little or no effect on the rhythmicity of postural tremor in Parkinson disease and essential tremor.
4. The within group relationships between amplitude HT, frequency HT and rhythmicity metrics are similar for ET and postural PDT but differ in PT. These relationships should be studied in patient populations with very mild ET and PDT and in control populations with enhanced physiological tremor.

## Supplementary Information

Supplementary Information.

## Data Availability

Tremor recordings will be shared in reasonable requests. Please contact Gregor Kuhlenbäumer and Günther Deuschl for details.
