# Supplementary material for: Diagnostic significance of rhythmicity in postural hand tremor
Source: Sci Rep. 2026 Jan 13;16:1954. doi: 10.1038/s41598-026-35257-3 (PMC12804936; doi:10.1038/s41598-026-35257-3)
Supplement: Supplementary file 1 — Supplementary Information. [file 41598_2026_35257_MOESM1_ESM.pdf]

SUPPLEMENTARY MATERIAL

Diagnostic significance of rhythmicity in postural hand tremor

Patricia Weede, Günther Deuschl, Rodger J. Elble, Robin Wolke, Gerhard Schmidt, and Gregor Kuhlenbäumer

Pearson correlation matrices of rhythmicity and tremor metrics for each diagnostic group and for PD and ET combined.

Supplementary Table S1: Pearson correlation matrix for physiologic tremor

		log2Amp_HT	Freq_HT	log2BW_HT	log2CTC_HT	log2TSI_HT	ApEn_HT
log2Amp_HT	Correlation coefficient<br>Significance Level P<br>n	1	-0.154<br>0.2909<br>49	-0.057<br>0.6979<br>49	0.072<br>0.6220<br>49	0.008<br>0.9574<br>49	-0.211<br>0.1453<br>49
Freq_HT	Correlation coefficient<br>Significance Level P<br>n	-0.154<br>0.2909<br>49	1	-0.223<br>0.1230<br>49	-0.057<br>0.6954<br>49	-0.153<br>0.2925<br>49	-0.080<br>0.5851<br>49
log2BW_HT	Correlation coefficient<br>Significance Level P<br>n	-0.057<br>0.6979<br>49	-0.223<br>0.1230<br>49	1	0.362<br>0.0106<br>49	0.560<br><0.0001<br>49	0.116<br>0.4288<br>49
log2CTC_HT	Correlation coefficient<br>Significance Level P<br>n	0.072<br>0.6220<br>49	-0.057<br>0.6954<br>49	0.362<br>0.0106<br>49	1	0.729<br><0.0001<br>49	0.303<br>0.0341<br>49
log2TSI_HT	Correlation coefficient<br>Significance Level P<br>n	0.008<br>0.9574<br>49	-0.153<br>0.2925<br>49	0.560<br><0.0001<br>49	0.729<br><0.0001<br>49	1	0.378<br>0.0075<br>49
ApEn_HT	Correlation coefficient<br>Significance Level P<br>n	-0.211<br>0.1453<br>49	-0.080<br>0.5851<br>49	0.116<br>0.4288<br>49	0.303<br>0.0341<br>49	0.378<br>0.0075<br>49	1

Supplementary Table S2: Pearson correlation matrix for essential tremor

		log2Amp_HT	Freq_HT	MT-HT coherence	log2Amp_MT	log2BW_HT	log2CTC_HT	log2TSI_HT	ApEn_HT
log2Amp_HT	Correlation coefficient<br>Significance Level P<br>n	1	-0.388<br><0.0001<br>133	0.402<br><0.0001<br>133	0.494<br><0.0001<br>133	-0.346<br><0.0001<br>133	-0.448<br><0.0001<br>133	-0.485<br><0.0001<br>133	-0.536<br><0.0001<br>133
Freq_HT	Correlation coefficient<br>Significance Level P<br>n	-0.388<br><0.0001<br>133	1	-0.041<br>0.6410<br>133	-0.264<br>0.0021<br>133	0.195<br>0.0242<br>133	0.014<br>0.8769<br>133	0.080<br>0.3587<br>133	0.253<br>0.0034<br>133
MT-HT coherence	Correlation coefficient<br>Significance Level P<br>n	0.402<br><0.0001<br>133	-0.041<br>0.6410<br>133	1	0.189<br>0.0293<br>133	-0.235<br>0.0064<br>133	-0.365<br><0.0001<br>133	-0.355<br><0.0001<br>133	-0.430<br><0.0001<br>133
log2Amp_MT	Correlation coefficient<br>Significance Level P<br>n	0.494<br><0.0001<br>133	-0.264<br>0.0021<br>133	0.189<br>0.0293<br>133	1	-0.110<br>0.2056<br>133	-0.118<br>0.1774<br>133	-0.151<br>0.0825<br>133	-0.190<br>0.0285<br>133
log2BW_HT	Correlation coefficient<br>Significance Level P<br>n	-0.346<br><0.0001<br>133	0.195<br>0.0242<br>133	-0.235<br>0.0064<br>133	-0.110<br>0.2056<br>133	1	0.578<br><0.0001<br>133	0.687<br><0.0001<br>133	0.407<br><0.0001<br>133
log2CTC_HT	Correlation coefficient<br>Significance Level P<br>n	-0.448<br><0.0001<br>133	0.014<br>0.8769<br>133	-0.365<br><0.0001<br>133	-0.118<br>0.1774<br>133	0.578<br><0.0001<br>133	1	0.865<br><0.0001<br>133	0.658<br><0.0001<br>133
log2TSI_HT	Correlation coefficient<br>Significance Level P<br>n	-0.485<br><0.0001<br>133	0.080<br>0.3587<br>133	-0.355<br><0.0001<br>133	-0.151<br>0.0825<br>133	0.687<br><0.0001<br>133	0.865<br><0.0001<br>133	1	0.649<br><0.0001<br>133
ApEn_HT	Correlation coefficient<br>Significance Level P<br>n	-0.536<br><0.0001<br>133	0.253<br>0.0034<br>133	-0.430<br><0.0001<br>133	-0.190<br>0.0285<br>133	0.407<br><0.0001<br>133	0.658<br><0.0001<br>133	0.649<br><0.0001<br>133	1

Abbreviations

Rhythmicity metrics:

ApEn: approximate entropy
TSI_HT: tremor stability index for hand tremor
CTC_HT: cycle-to-cycle frequency variability for hand tremor
BW_HT: half-peak-power bandwidth for the power spectral peak of hand tremor

Tremor metrics:

Amp_HT: amplitude of hand tremor (accelerometry)
Amp_MT: amplitude of muscle tremor (electromyography)
Freq_HT: frequency of hand tremor
MT-HT coherence: coherence between muscle tremor and hand tremor

Supplementary Table S3: Pearson correlation matrix for Parkinson disease

		log2Amp_HT	Freq_HT	MT-HT coherence	log2Amp_MT	log2BW_HT	log2CTC_HT	log2TSI_HT	ApEn_HT
log2Amp_HT	Correlation coefficient<br>Significance Level P<br>n	1	-0.465<br><0.0001<br>78	0.442<br>0.0001<br>78	0.758<br><0.0001<br>78	-0.250<br>0.0275<br>78	-0.589<br><0.0001<br>78	-0.655<br><0.0001<br>78	-0.559<br><0.0001<br>78
Freq_HT	Correlation coefficient<br>Significance Level P<br>n	-0.465<br><0.0001<br>78	1	-0.244<br>0.0316<br>78	-0.269<br>0.0172<br>78	0.203<br>0.0747<br>78	0.181<br>0.1130<br>78	0.231<br>0.0423<br>78	0.232<br>0.0409<br>78
MT-HT coherence	Correlation coefficient<br>Significance Level P<br>n	0.442<br>0.0001<br>78	-0.244<br>0.0316<br>78	1	0.319<br>0.0045<br>78	-0.376<br>0.0007<br>78	-0.407<br>0.0002<br>78	-0.582<br><0.0001<br>78	-0.440<br>0.0001<br>78
log2Amp_MT	Correlation coefficient<br>Significance Level P<br>n	0.758<br><0.0001<br>78	-0.269<br>0.0172<br>78	0.319<br>0.0045<br>78	1	-0.113<br>0.3226<br>78	-0.486<br><0.0001<br>78	-0.501<br><0.0001<br>78	-0.399<br>0.0003<br>78
log2BW_HT	Correlation coefficient<br>Significance Level P<br>n	-0.250<br>0.0275<br>78	0.203<br>0.0747<br>78	-0.376<br>0.0007<br>78	-0.113<br>0.3226<br>78	1	0.507<br><0.0001<br>78	0.588<br><0.0001<br>78	0.333<br>0.0029<br>78
log2CTC_HT	Correlation coefficient<br>Significance Level P<br>n	-0.589<br><0.0001<br>78	0.181<br>0.1130<br>78	-0.407<br>0.0002<br>78	-0.486<br><0.0001<br>78	0.507<br><0.0001<br>78	1	0.829<br><0.0001<br>78	0.588<br><0.0001<br>78
log2TSI_HT	Correlation coefficient<br>Significance Level P<br>n	-0.655<br><0.0001<br>78	0.231<br>0.0423<br>78	-0.582<br><0.0001<br>78	-0.501<br><0.0001<br>78	0.588<br><0.0001<br>78	0.829<br><0.0001<br>78	1	0.627<br><0.0001<br>78
ApEn_HT	Correlation coefficient<br>Significance Level P<br>n	-0.559<br><0.0001<br>78	0.232<br>0.0409<br>78	-0.440<br>0.0001<br>78	-0.399<br>0.0003<br>78	0.333<br>0.0029<br>78	0.588<br><0.0001<br>78	0.627<br><0.0001<br>78	1

Supplementary Table S4: Pearson correlation matrix for essential tremor and Parkinson tremor combined

		log2Amp_HT	Freq_HT	MT-HT coherence	log2Amp_MT	log2BW_HT	log2CTC_HT	log2TSI_HT	ApEn_HT
log2Amp_HT	Correlation coefficient<br>Significance Level P<br>n	1	-0.428<br><0.0001<br>211	0.420<br><0.0001<br>211	0.602<br><0.0001<br>211	-0.321<br><0.0001<br>211	-0.478<br><0.0001<br>211	-0.545<br><0.0001<br>211	-0.538<br><0.0001<br>211
Freq_HT	Correlation coefficient<br>Significance Level P<br>n	-0.428<br><0.0001<br>211	1	-0.123<br>0.0744<br>211	-0.271<br>0.0001<br>211	0.202<br>0.0032<br>211	0.081<br>0.2424<br>211	0.148<br>0.0311<br>211	0.246<br>0.0003<br>211
MT-HT coherence	Correlation coefficient<br>Significance Level P<br>n	0.420<br><0.0001<br>211	-0.123<br>0.0744<br>211	1	0.239<br>0.0004<br>211	-0.284<br><0.0001<br>211	-0.373<br><0.0001<br>211	-0.437<br><0.0001<br>211	-0.433<br><0.0001<br>211
log2Amp_MT	Correlation coefficient<br>Significance Level P<br>n	0.602<br><0.0001<br>211	-0.271<br>0.0001<br>211	0.239<br>0.0004<br>211	1	-0.117<br>0.0888<br>211	-0.263<br>0.0001<br>211	-0.300<br><0.0001<br>211	-0.278<br><0.0001<br>211
log2BW_HT	Correlation coefficient<br>Significance Level P<br>n	-0.321<br><0.0001<br>211	0.202<br>0.0032<br>211	-0.284<br><0.0001<br>211	-0.117<br>0.0888<br>211	1	0.542<br><0.0001<br>211	0.642<br><0.0001<br>211	0.379<br><0.0001<br>211
log2CTC_HT	Correlation coefficient<br>Significance Level P<br>n	-0.478<br><0.0001<br>211	0.081<br>0.2424<br>211	-0.373<br><0.0001<br>211	-0.263<br>0.0001<br>211	0.542<br><0.0001<br>211	1	0.847<br><0.0001<br>211	0.626<br><0.0001<br>211
log2TSI_HT	Correlation coefficient<br>Significance Level P<br>n	-0.545<br><0.0001<br>211	0.148<br>0.0311<br>211	-0.437<br><0.0001<br>211	-0.300<br><0.0001<br>211	0.642<br><0.0001<br>211	0.847<br><0.0001<br>211	1	0.639<br><0.0001<br>211
ApEn_HT	Correlation coefficient<br>Significance Level P<br>n	-0.538<br><0.0001<br>211	0.246<br>0.0003<br>211	-0.433<br><0.0001<br>211	-0.278<br><0.0001<br>211	0.379<br><0.0001<br>211	0.626<br><0.0001<br>211	0.639<br><0.0001<br>211	1
